# The Impact of Caregiving on Informal Caregivers of People with Dementia: Family Functioning, Burden, and Burnout

**DOI:** 10.1007/s10880-024-10052-2

**Published:** 2024-10-25

**Authors:** Rute Brites, Tânia Brandão, Odete Nunes, João Hipólito, Catarina Tomé Pires

**Affiliations:** 1 CIP-UAL, Psychology Department, Universidade Autónoma de Lisboa, Lisbon, Portugal; 2 William James Center for Research, ISPA–Instituto Universitário, Lisbon, Portugal

**Keywords:** Burnout, Burden, Informal caregiving, Family functioning, Dementia, Neuropsychiatric symptoms

## Abstract

Caregiving is a complex occupation, with a significant impact for informal caregivers (IC). Stress-process models propose a framework that considers that this impact depends on primary and secondary stressors, but also on the IC situation appraisal. This work aimed to verify: whether being, or not, an IC of an individual with dementia influenced the relationship between family functioning and burnout; the association between neuropsychiatric symptoms of the individual with dementia and IC burnout and whether the IC burden and perceived family functioning had a mediating role in such relationship. This cross-sectional study investigated differences in family functioning and its association with burnout between IC and non-IC. For IC specifically, the study examined a mediation model to explore the possibility of neuropsychiatric symptoms indirectly affecting IC burnout through the impact on family functioning and burden. Measures included the Copenhagen Burnout Inventory, the McMaster Family Assessment Device, the Neuropsychiatric Inventory, and the Zarit Burden Interview. Results showed an association between poorer family function and high burnout, specifically in IC. They also showed that burden mediated the relationship between neuropsychiatric symptoms and burnout. The findings offer a significant contribution to the growing knowledge about the relationship between stressors associated with informal caregiving in dementia context, such as neuropsychiatric symptoms and its outcomes, like burnout.

## Introduction

With increasing global life expectancy, dementia cases are rising rapidly, projected to reach 139 million by 2050 from 55 million cases in 2019 (WHO, 2021). Europe is experiencing a similar trend, with dementia diagnoses increasing in every country (Tokovska et al., 2022). The Alzheimer’s Yearbook (2019) estimates around 8 million people in the European Union (EU) live with dementia, a number expected to grow, straining healthcare services due to rising care needs.

The rising demand for healthcare has a significant financial and personal cost. According to Eurocarers, IC provide 80% of care in Europe, and the economic value of this unpaid service is estimated at 50–90% of the total cost of formal long-term care in the EU. IC are those who “provide—usually—unpaid care to someone with a chronic illness, disability or other long-lasting health or care need, outside a professional or formal framework” (Eurocarers, 2023).

In most cases, informal caregiving is a challenging occupation, with a significant psychological impact for those who undertake this role. Studies describe these consequences, such as fatigue, stress, depression, anxiety, hostility, anger, frustration, guilt, fear, loneliness, pessimism, or decreased self-esteem (Lage, 2005; Liu & Gallagher-Thompson, 2009; Peeters et al., 2010), and burden (Gérain & Zech, 2019). Regarding IC of individuals with dementia, studies have consistently shown that they experience high levels of burden, depression, anxiety, distress, higher physical morbidity (Brites et al., 2020; Collins & Kishita, 2019; Joling et al., 2018; Ulstein et al., 2007), social isolation, physical ill-health, financial hardship (Brodati & Donkin, 2009), and poor quality of life (Frias et al., 2020).

For these IC, challenges are related to the cognitive impairment of the individual being cared, their violent and disruptive behavior, their functional difficulties, coupled with a lack of knowledge about dementia. These factors result in extensive caregiving responsibilities for the caregiver and, as the condition progresses, full-time care (Ali & Bokharey, 2016; Zahed et al., 2020).

Informal caregiving is a complex and enduring stressful situation influenced by distinct variables. Revenson et al.’s (2016) framework views caregiving as a stressor that poses significant health challenges. The IC assesses this situation, perceiving it as either stressful or challenging, leading to specific negative or positive outcomes.

Some authors have tried to identify various types of stressors and integrate them into explanatory models (Table 1).

**Table 1:** Explanatory Models of the Caregiving Stress-Process

Authors	Primary Stressors (associated with the dementia)	Secondary Stressors (implications of primary stressors)	Outcomes
Gérain & Zech, 2019*	Time since the start of caregiving Duration and/or intensity of care Functional impairment Intensity of symptoms	Disturbance in daily life Reduced social life Loss of friends “Giving up” important things	Burden Burnout
Schultz & Martire, 2004*	Problem behaviors Disability Loss	Family conflict Work difficulties	Perceived stress
Pearlin et al., 1990*	Cognitive status Problematic behavior ADL dependency	Family conflict Job-Caregiving conflict Economic problems Constriction of social fife	Depression Anxiety Irascibility Cognitive disturbance Physical health Yielding of role
Zarit et al., 1980	Behavioral problems Deteriorating cognitive ability Increased impairment in activities of daily living (ADL)	Impact on social life Job stressors Family problems Lack of social support	Burden

*Given the complexity of the models, we have chosen to present only the part of the models that relates to our research topic, highlighting the primary and secondary stressors

One of the main primary stressors identified is related to the behavioral and psychological aspects of the disease itself (Connors et al., 2020; Delfino et al., 2021; Farcnik & Persyko, 2002; Lian et al., 2017; van der Lee et al., 2014). Aggression, agitation, night-time wandering, psychosis, mood disturbance, irritability, hallucinations, and depression were some of the behaviors strongly associated with low mental health outcomes (Gaugler et al., 2005; Torrisi et al., 2017), mainly burden (Torrisi et al., 2017; Yıldızhan et al., 2019), but also burnout (Truzzi et al., 2012; Yıldızhan et al., 2019).

Family caregivers of older adults with disabilities face a multitude of physical, psychological, social, and financial challenges, known as a burden (George & Gwyther, 1986). This burden is influenced by their perception of activities and stressors, shaped by various psychosocial factors (Etters et al., 2008).

Zarit et al. (1986) proposed two “approaches” on burden: objective burden, related to family, financial and supervision difficulties, and subjective burden, related to the IC emotional response to caregiving. More recently, Zarit and Zarit (2015) clarified that subjective burden should be considered as the personal experience of the caregiver. It can be described as the subjective evaluation of the stress that the caring situation represent (Galiatsatos et al., 2017). In this sense, some authors (Gérain & Zech, 2022; Revenson et al., 2016) consider that subjective burden appears as a key mediator between the demands of caregiving and caregiving outcomes, such as burnout.

Burnout is an occupational syndrome that occurs in response to chronic and interpersonal stressors in the work environment (Truzzi et al., 2012). Gérain and Zech (2019) consider that it can be applied to the caregiving context.

Onwumere et al. (2017) found that the caregiver’s appraisals (about themselves, the illness, and their own coping skills) play an important role in their functioning and well-being, regardless of the symptomatology of the individual with dementia. Thus, caregiver burnout can be understood as a response to the stress that arises from the demanding nature of the caregiving context (Gérain & Zech, 2019).

Several studies have reported significant levels of burnout in the context of informal caregiving (Gérain & Zech, 2021; Takai et al., 2009; Yıldızhan et al., 2019), mainly related to primary stressors such as cognitive, behavioral, and emotional changes in the individual with dementia (Hiyoshi-Taniguchi et al., 2018; van den Heuvel et al., 2001). However, less is known about the processes involved in such associations. Gérain and Zech (2019) emphasize the importance of studying not only primary stressors but also their implications (secondary stressors) and IC’s perceptions (appraisal). Several caregiving stress-process models (e.g., Gérain & Zech, 2019; Pearlin et al., 1990; Schultz & Martire, 2004; Zarit et al., 1980) identify secondary stressors (or secondary role strains) related to burden, including negative effects on social, professional, and family life.

Considering family life, several studies have examined interpersonal variables (between the IC and the individual with dementia), such as relationship continuity (e.g., Riley et al., 2018), relationship satisfaction (Steadman et al., 2007) or dyadic relational resources like mutuality, preparedness, and predictability (Yang et al., 2014). However, less is known about the overall family aspects such as family functioning, described as the way in which the family members interact, react to, and treat other family members (Olson & Gorall, 2011). Spitznagel et al. (2006) highlighted the association between overall family functioning, the desire to institutionalize the individual with dementia and caregiver’s burden. Other studies described a similar pattern, with caregivers with poorer family functioning showing higher levels of strain and burden (Chiou et al., 2009; Liu et al., 2016; Marinho et al., 2022). In contrast, some authors have highlighted the importance of family support for a positive appraisal of informal caregiving (Lampley-Dallas et al., 2001) and the reduction of caregivers’ burden (Lindeza et al., 2020). Thus, given the potential negative impact of the dementia caregiving in the family (Oh et al., 2020), it is relevant to ascertain its mediator role.

Research has consistently linked the behavioral and psychological aspects of the dementia to IC mental health outcomes (Brandão et al., 2024; Delfino et al., 2021; van der Lee et al., 2014; Torrisi et al., 2017; Truzzi et al., 2012), but the processual relationship between these stressors and caregiver outcomes remains unexplored. This study aims to: (1) examine caregiving status (i.e., being an informal caregiver or a non-caregiver) as a moderator of the association between family functioning and burnout and (2) test a novel mediation model examining the mediating role of family functioning and burden on the association between neuropsychiatric symptoms and burnout.

## Methods

In this cross-sectional study, participants answered to several measures to obtain data related to caregiving and its impact. We hypothesized that:

### H1

More positive family functioning is associated with lower levels of burnout;

### H2

The association between family functioning and burnout is different depending on whether one belongs to the IC group or not, i.e., caregiving status acts as a moderating variable;

### H3

For IC, the neuropsychiatric symptoms experienced by the individual with dementia are associated with more burnout;

### H4

The level of burden of IC mediates the association between neuropsychiatric symptoms and burnout;

### H5

The family functioning perceived by the IC mediates the association between neuropsychiatric symptoms and burnout.

## Procedure

The study was approved by the Ethical Committee of the Psychology Research Centre (CIP-UAL) and by the hospitals’ Clinical Research Ethics Committees. IC were approached during their routine visits to the hospital consultation for the individual with dementia. After describing the objectives of the study to the participants, their written informed consent was obtained. Assessment was completed during their appointment taking on average 30 min. IC were included if they were the main caregivers of an individual with dementia and did not benefit from psychological support.

Regarding non-IC, research assistants recruited them in the community and explained the aim of the study, emphasizing the importance of not being a IC for a vulnerable person. Written informed consent was obtained. Participants with recent caregiving experience, those receiving psychological or psychiatric care, and those with serious illness or current life difficulties were excluded. No incentives or monetary compensation were offered for participation.

## Participants

The sample comprised 162 adult participants, including 78 IC and 84 non-IC.

Considering the ICs’ sociodemographic characteristics, their age ranged from 19 to 89 years (M = 64.84, SD = 13.32). Most of them were women, married and the spouse of the individual with dementia. Almost half of the participants had basic education (primary school), followed by those who completed high school. More than a half of the ICs were retired.

Regarding information about the individuals with dementia, in most cases the diagnosis was Alzheimer disease. On average, diagnosis was made 5.52 years ago (SD = 4.34), ranging from 6 months to 19 years. Table 2 presents all sociodemographic and clinical data.

**Table 2:** Sociodemographic Characteristics of the Participants (IC and non-IC)

		IC (*N* = 78)		Non-IC (*N* = 84)	
		*n*	%	*n*	%
Sex	Female	56	71.8	62	73.8
	Male	22	28.2	22	26.2
Education					
	Knows how to read and write	1	1.3	-	-
	Primary school	32	41.6	14	16.9
	Middle school	13	16.9	16	19.2
	High school	18	23.4	20	24.1
	University	8	10.4	33	39.8
	Other	6	7.8	-	-
Professional status					
	Retired	45	57.7	23	27.4
	Employed	20	25.6	50	59.5
	Unemployed	10	12.8	6	7.2
	Without information	3	3.8	5	6.0
Marital status					
	Married/civil partner	56	71.8	51	60.7
	Separated/ divorced	13	16.7	19	22.6
	Single	6	7.7	6	7.1
	Widower	3	3.8	8	9.5
Relationship with the individual with dementia (only IC)				-	-
	Spouse	42	53.8	-	-
	Adult children	27	34.6	-	-
	Grandchild	2	2.6	-	-
	Nephew	2	2.6	-	-
	Daughter-in law	1	1.3	-	-
	Other	2	2.6	-	-
	Non-related	2	2.6	-	-
Diagnosis (individual with dementia) (only IC)					
	Alzheimer disease	49	62.8	-	-
	Vascular dementia	6	7.7	-	-
	Lewy body dementia	4	5.1	-	-
	Frontotemporal dementia	2	2.6	-	-
	Other	17	21.8	-	-

With respect to the non-IC, their age ranged from 50 to 83 years (M = 61.07, SD = 7.50). Most were women and married. The majority had completed high school or had a university degree, and were employed.

## Measures

### Sociodemographics

Participants were asked to provide information about their age, sex, education level, professional and marital status, relationship to the individual with dementia and the diagnosis of that individual.

### Family Functioning

Family functioning was assessed with the McMaster Family Assessment Device (FAD) (Almeida et al., 2020; Epstein et al., 1983). This questionnaire assesses the family structure, organization, and transactional models. The original version of FAD is composed by 60 items, organized into seven subscales (Epstein et al., 1983). In the present study, only the 12-item general functioning subscale was used, assessing overall family functioning perception (Almeida et al., 2020; Ryan et al., 2005) (example: “*Making decisions is a problem for our family*”). The items are answered on a Likert-type scale (1 = “Strongly Disagree” to 4 = “Strongly Agree”). In this study, the internal consistency was good, both for IC (*α* = .81) and non-IC (*α* = .84) (α total sample = .81).

### Burnout

Burnout was examined with The Copenhagen Burnout Inventory (CBI) (Fonte, 2011; Kristensen et al., 2005). This scale comprises 19 items, organized into three subscales—personal burnout, work-related burnout, and client-related burnout (Kristensen et al., 2005). In the present study, only the 6-item personal burnout subscale was used (example: “*How often are you emotionally exhausted?*”). Items are scored through a Likert-type scale ranging from 1 (never) to 5 (always) (Fonte, 2011). In the present study, this subscale presented excellent internal consistency (*α* IC = .92, *α* = non-IC = .82, *α* total sample = .90).

### Neuropsychiatric Symptoms

The Neuropsychiatric Inventory (NPI) assesses the presence of neuropsychiatric symptoms in people with dementia (Cummings et al., 1994; Kaufer et al., 2000; Espírito-Santo et al., 2010). It includes 12 questions referring to 12 domains of neuropsychiatric symptoms (delirium, hallucinations, agitation, depression/dysphoria, anxiety, euphoria/relation, apathy/indifference, disinhibition, irritability/lability, motor behavior, sleep, and appetite) (Reis, 2011).

This assessment of symptoms refers to the previous month, and the IC is asked to reflect on the presence of these symptoms, indicating Yes (if present) or No (if absent) (Kaufer et al., 2000). When the answer is affirmative, the IC must indicate the severity of that symptom along a Likert scale from 1 to 3 (1—mild; 2—moderate; 3—severe) and how disturbed they are by that symptom, on a Likert scale from 0 to 5 (0—no upsetting; 5—extremely upsetting) (Kaufer et al., 2000). In each item, the severity is multiplied by the degree of disturbance. The final score is the sum of the results for all items. In the original version, Cronbach’s alpha was .88 (Cummings et al., 1994). In the present study, the reliability was good (*α* = .81).

### Burden

Burden was assessed with the Zarit Burden Interview (Pereira et al., 2008; Zarit et al., 1980). This self-report questionnaire is composed of 22-items (example: “*Do you feel that because of the time you spend with your relative that you don’t have enough time for yourself?*”), rated on a four‐point rating scale ranging from 0 (never) to 4 (nearly always), with higher scores indicating greater burden (range 0–88) (Gonçalves-Pereira & Zarit, 2014). For this study, good internal consistency was found (*α* = .87).

### Data Analysis

Data were analyzed using SPSS (v. 26; IBM, SPSS Inc.). Descriptive statistics and bivariate Pearson correlations were calculated. Independent samples t-tests were conducted to explore differences between IC and non-IC, concerning study variables. Moderation and mediation models were tested using SPSS Process Macro v. 4.0 (Hayes, 2022).

The moderation model (PROCESS model 1) included an independent variable (i.e., family functioning), a dependent variable (i.e., burnout), and one dichotomous moderator (i.e., being or not an IC). Direct and conditional effects of the independent variable were examined, using bootstrapping with 5000 bootstrap samples. Conditional effect was considered significant if the 95% bias corrected confidence intervals did not include zero.

The serial mediation model (PROCESS model 6) included an independent variable (i.e., neuropsychiatric symptoms), a dependent variable (i.e., burnout), and two mediators (family functioning and burden), operating in serial. Direct, indirect, and specific indirect effects (contrasts) were examined, using bootstrapping with 5000 bootstrap samples. Confidence interval for all possible pairwise comparisons between specific indirect effects were analyzed to compare them. Indirect effects and specific indirect effects were considered significant when the 95% confidence intervals did not include zero.

## Results

When comparing IC and non-IC, there were no significant differences in sociodemographic characteristics with regard to sex (Χ^2^_(1)_ = 0.08, *p* = .77) and age (men, *t*_(29.8)_ = 1.91, *p* = .07; women, *t*_(86.04)_ = 1.37, *p* = .17).

Regarding study variables, IC reported significantly higher burnout scores compared to non-IC. There were no statistically significant differences in family functioning scores between the two groups. Table 3 provides information about the burnout and family functioning scores for both groups.

**Table 3:** Descriptive Statistics for IC and non-ICs Concerning Family Functioning and Burnout, and Group Comparison

Variable	IC		Non-IC		Difference
	M	SD	M	SD	
Family functioning	3.13	0.53	3.16	0.52	*t*_(160)_ = − 0.33
Burnout	3.50	1.09	2.97	0.68	***t***_**(160)**_ = 3.65***

***p < .001. Significant differences are flagged in bold

Pearson bivariate correlations demonstrated a negative association between family functioning and burnout. Higher levels of family functioning were associated with lower levels of burnout, and vice-versa.

Specifically, regarding the impact of the neuropsychiatric symptoms of the individual with dementia on the IC, there were positive significant associations between neuropsychiatric symptoms and burden, and burnout. There was a significant negative association between burden and family functioning and a significant positive association between burden and burnout. A significant negative association between family functioning and burnout was presented. Descriptive statistics and Pearson bivariate correlations are presented in Table 4.

**Table 4:** Descriptive Statistics and Bivariate Correlations among Study Variables, in the IC group (*n* = 78)

	M (SD)	1	2	3	4
1. NPS	42.15 (31.26)	-	.35^**^	− .19	.39^**^
2. Burden	20.31 (10.40)		-	− .45^**^	.73^***^
3. Family functioning	3.13 (0.53)			-	− .28^***^
4. Burnout	3.50 (1.09)				-

**p < 01; *** *p* < .001; *NPS* Neuropsychiatric symptoms

To test the hypothesis that being an IC (or not) moderates the relationship between family functioning and burnout, a simple moderation analysis was conducted. These variables accounted for a significant amount of variance in burnout, R^2^ = 0.186, *F* (3, 158) = 12.03, *p* < .001. Specifically, results showed that the interaction term between family functioning and being or not an IC accounted for a significant proportion of the variance in burnout, Δ*R*2 = 0.03, Δ*F* (1, 158 = 5.80, *p* = .017).

**Fig. 1:**
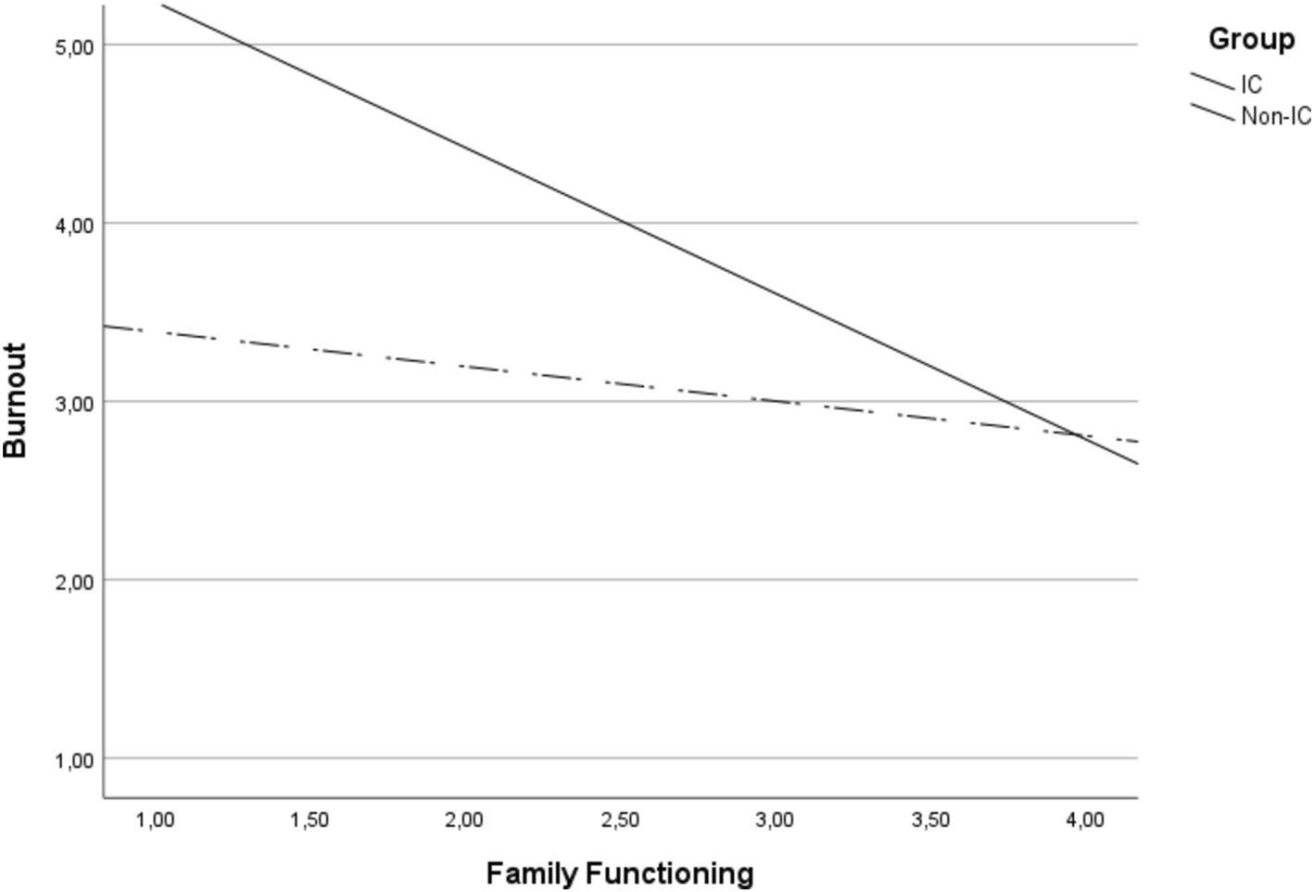
Interaction plot: association between family functioning and burnout in IC and non-IC

The analysis of the interaction plot (Fig. 1) showed that in both groups, when family functioning was high, burnout levels were low. On the contrary, when family functioning was low, burnout levels were higher, especially in the group of IC, where the interaction was significant. Specifically, in the group of IC, interaction with family functioning had an effect of—0.818 [CI = − 1.18, − 0.45]. In the group of non-caregivers, interaction with family functioning had an effect of—0.194 [CI = − 0.55, 0.16]. Only in the group of IC was the interaction statistically significant, *t* = − 1.07, *p* < .001.

After, a mediation model with multiple mediators was tested. Burden and family functioning were introduced serially as mediators of the association between neuropsychiatric symptoms and burnout (see Table 5).

**Table 5:** Direct, indirect, specific indirect effects (contrasts) and total effects of neuropsychiatric symptom on burnout through family functioning and burden

	Direct Effect			Indirect Effect*		Total Effect		
	B (SE)	95%CI	*p*	B (SE)	95%CI	B (SE)	95%CI	*p*
NPS- > FF	− 0.003 (0.002)	(− 0.007, 0.001)	.14	-	-	− 0.003 (0.002)	(− 0.007, 0.001)	.14
NPS- > Burden	0.083 (0.034)	(0.014, 0.150)	** < .05**	-	-	**0.083 (0.034)**	**(0.014, 0.150)**	** < .05**
FF- > Burnout	0.028 (0.233)	(− 0.437, 0.495)	.90	-	-	0.028 (0.233)	(− 0.437, 0.495)	.90
Burden- > Burnout	0.073 (0.012)	(0.048, 0.099)	** < .001**	-	-	**0.073 (0.012)**	**(0.048, 0.099)**	** < .001**
NPS- > FF- > Burnout (Ind1)	0.006 (0.003)	(− 0.001, 0.013)	.10	− 0.00 (0.001)	(− 0.002,0.002)	**0.014 (0.004)**	**(0.006, 0.023)**	** < .01**
NPS- > Burden- > Burnout (Ind2)				**0.006 (0.003)**	**(0.001, 0.013)**			
NPS- > FF- > Burden- > Burnout (Ind3)				0.003 (0.002)	(− 0.000, 0.006)			
Contrast 1 (Ind 1–Ind2)				− **0.006 (0.003)**	**(**− **0.014, -0.001)**			
Contrast 2 (Ind 1–Ind3)				− 0.003 (0.002)	(− 0.008, 0.000)			
Contrast 3 (Ind 2–Ind3)				0.004 (0.003)	(− 0.003, 0.011)			
R^2^ = 15%								

Bold font indicates significant effects (*p* < .05). B = unstandardized regression coefficients; SE = standard error; 95%CI bootstrapped 95% confidence Intervals; *p* = *p*-values. *Indirect effects and contrasts were considered significant in the presence of 95% confidence intervals not including zero. *NPS* neuropsychiatric symptoms; *FF* family functioning; *Ind* Indirect Effect

Although there was not a direct effect of neuropsychiatric symptoms on burnout, the analysis of the confidence intervals showed that there was a significant indirect effect through burden (controlling for family functioning), but not through family functioning. Burden mediated this association, which means that higher neuropsychiatric symptoms were associated to more burden which, in turn, was associated with higher burnout.

The specific indirect effect of neuropsychiatric symptoms on burnout through burden is significantly different from the specific indirect effect through family functioning.

## Discussion

The current study examined the association between family functioning and burnout among participants categorized by caregiving status (IC or non-IC). It also explored whether family functioning and burden mediated the relationship between the neuropsychiatric symptoms of the individual with dementia and IC perceived burnout.

Our results confirmed that positive family functioning was associated with lower burnout, thus confirming H1. Furthermore, they also confirmed our second hypothesis (H2) as it was found that caregiving status moderated this relationship, with a significant association only for IC. Low family functioning is associated with higher levels of burnout in IC.

These findings are in line with previous studies that have found an association between poor family functioning and negative IC outcomes such as strain and burden, both in the context of dementia care (Liu et al., 2016; Spitznagel et al., 2006) and other care contexts (Chiou et al., 2009). Other studies, in contrast, have highlighted the importance of family support for a positive evaluation of informal caregiving (Lampley-Dallas et al., 2001) and the positive impact of low family conflict on caregiver experiences (Lin et al., 2012), which agrees with the results now presented.

According to Vasileiou et al. (2017), the caregiving situation leads to an inevitable reorganization of family relationships, with a negative impact on IC. Although informal care is largely an individual endeavor, the feeling of shared difficulties and support from family members seems to be a protective aspect against its negative consequences.

Additionally, this study provides new evidence regarding the differences in psychological functioning, specifically burnout, between IC of individuals with dementia and non-IC. The current findings are consistent with the review of Gérain and Zech (2021), which showed that informal caregiving (in contexts other than dementia) poses a risk of burnout by adding an additional weight to the “shoulders” of IC, when compared with non-IC.

The results of this study also provide evidence to support that burden, but not family functioning, mediates the association between the neuropsychiatric symptoms of the individual with dementia and the burnout perceived by the IC, allowing H3 and H4 to be confirmed, but not H5.

This study has shown that burden plays an important role in the association between the behavioral and psychological aspects of dementia and the mental health of the IC, namely burnout. In fact, neuropsychiatric symptoms are associated with burnout through burden. Directly, these symptoms are not predictors of burnout. Onwumere et al. (2017) highlighted the role of the IC assessment of their well-being, regardless of the symptoms of the individual with dementia. In line with the findings of Lee and Singh (2010), our results strongly imply that burnout is the result of a subjective process of evaluation of the situation on the part of the IC, and not a product of the fatigue and tension resulting from the task itself. With regard to family functioning, our results seem to indicate that the IC perception of this aspect is not related to the symptoms presented by the individual with dementia. This leads us to hypothesize that it is a preponderant variable for the IC individual functioning, probably associated with their needs, feelings, and expectations, in line with other studies (Schulz & Martire, 2004; Shanley et al., 2011). It may not depend on the severity of the symptoms of the individual with dementia, but essentially on the IC experience of providing care. Some authors point out that the relationship between the IC and their family is affected by the fact that the IC takes responsibility for the caregiving of the individual with dementia and its consequences (Cascioli et al., 2008; Shanley et al., 2011). Thus, their assessment of family functioning can result from family members' well-known need for emotional support (Schulz & Martire, 2004) or from a feeling of loneliness associated with the perception that the family has moved away from them (Shanley et al., 2011).

Family functioning has been studied in relation to its association with IC outcomes, such as burden and strain (Chiou et al., 2009; Lindeza et al., 2020; Liu et al., 2016), but there is a lack of evidence on the aspects of the informal caregiving situation that can affect this functioning.

Our results highlight the potential of good family functioning as a protective variable for IC psychological functioning (Lampley-Dallas et al., 2001; Lindeza et al., 2020). Nordtug et al. (2021) emphasized the usefulness of the IC talking to people in their primary relationships. Sharing experiences and challenges in care helped them feel seen, gain understanding and receive help and support. In our opinion, this can apply in a similar way to family members. It is likely that if the IC feels supported by family members, they will have a more positive caregiving experience (Lampley-Dallas et al., 2001). On the other hand, if they feel abandoned or devalued, they may evaluate the caregiving experience more negatively (Shanley et al., 2011), with more negative consequences for their psychological functioning.

## Limitations

Despite the valuable contribution of this work, certain limitations of this study should be addressed in future research. The study have a cross-sectional design, which limits any conclusions about causality, other than in theoretical terms. Furthermore, data are based on self-report measures. In addition, the study only included IC of individuals with dementia, preventing to generalize these results to other care contexts. Additionally, the type of family (or other) relationship the IC had with the individual with dementia was not controlled for. The IC group was essentially composed by individuals with close emotional ties to the individual with dementia, which may have influenced the assessment of the individual impact of care provision. Finally, the family functioning and burnout measures assess general characteristics. They were not specifically designed to assess caregiving. The questions do not refer to the caregiving context and do not consider the existence of a diseased individual in the family who requires permanent care and whose situation is expected to worsen.

### Theoretical and Practical Implications

These results suggest several theoretical and practical implications. In theoretical terms, our findings give support to the stress-process models (Farcnik & Persyko, 2002; Gérain & Zech, 2019; Pearlin et al., 1990) that consider the existence of primary stressors (in this case, the neuropsychiatric symptoms of the individual with dementia), secondary stressors (in this case, family functioning), their value for the IC (the “appraisal,” here conceptualized as burden), and outcomes (in our study, burnout). In fact, they support the idea that the impact of primary stressors on IC functioning is influenced by numerous variables, and that their subjective appraisal is preponderant, in this relationship. At the same time, it raises questions about the family functioning variable, which deserves further exploration within this context.

In practical terms, these findings have some potential intervention implications. They stress the idea that interventions targeting IC should be tailored, considering their internal resources and vulnerabilities. The information offered is relevant, in the sense of providing IC with greater knowledge about the disease and its consequences. However, psychological work is fundamental to make them aware (1) of the various variables that influence their state of mental health and (2) of the way in which they appraise the care situation and change it, when necessary.

Regarding caregiving research, this study emphasizes the importance of the caregiver’s appraisal of the situation. It goes beyond the presence of stress factors and emphasizes the central role of this subjective process. It also identifies family functioning as a factor that influences the caregiver's well-being. However, it emphasizes the need for more research into their specific role in the stress process of IC of people with dementia.

Future research should continue to examine the impact of informal caregiving and the role of internal IC resources as protective or risk variables, using a process approach and longitudinal design. Several studies indicate that burnout is a potential predictor of other mental health outcomes such as psychological distress, depression, anxiety, quality of life among others (Alves et al., 2019; Gérain & Zech, 2022; Götze et al., 2015), so these hypotheses should be better explored. Measures should be developed to accommodate the specificities of this group (e.g., burnout and family functioning). In addition, psychophysiological measures could be used alongside psychological ones to capture specific changes in IC functioning over time, considering worsening dementia symptoms and increased dependence on the individual with dementia.

This study advances our understanding of the relationship between IC stressors and outcomes. We hope it will stimulate further investigation in this area as the aging European population leads to an increase in IC, who are at high risk for mental health problems.

## Data Availability

Data is available under reasonable request by emailing the first author.
